# Anticipating the emergence of infectious diseases

**DOI:** 10.1098/rsif.2017.0115

**Published:** 2017-07-05

**Authors:** Tobias S. Brett, John M. Drake, Pejman Rohani

**Affiliations:** 1 Odum School of Ecology, University of Georgia, Athens, GA, USA; 2 Center for the Ecology of Infectious Diseases, University of Georgia, Athens, GA, USA; 3 Department of Infectious Diseases, University of Georgia, Athens, GA, USA

**Keywords:** modelling of infectious disease, epidemiology, complex systems, early-warning signals

## Abstract

In spite of medical breakthroughs, the emergence of pathogens continues to pose threats to both human and animal populations. We present candidate approaches for anticipating disease emergence prior to large-scale outbreaks. Through use of ideas from the theories of dynamical systems and stochastic processes we develop approaches which are not specific to a particular disease system or model, but instead have general applicability. The indicators of disease emergence detailed in this paper can be classified into two parallel approaches: a set of early-warning signals based around the theory of critical slowing down and a likelihood-based approach. To test the reliability of these two approaches we contrast theoretical predictions with simulated data. We find good support for our methods across a range of different model structures and parameter values.

## 1. Introduction

Tipping points—where small changes in circumstances precipitate dramatic shifts in state—are a feature of many natural systems. These sudden transitions can have devastating consequences, for example, irreversible climate change [1] or ecological collapse [2]. Forewarning, with sufficient time to act, is of clear importance.

In principle, careful scientific research and detailed mechanistic understanding could lead to the formulation of predictive models capable of anticipating such transitions. However the combination of nonlinearity, non-stationarity, noise and data availability is a barrier to mechanistic modelling for many natural systems. There has been a trend towards looking for alternative methods of anticipating transitions, which do not rely on an empirically validated model [3]. One promising avenue is the development of early-warning signals (EWS) [4], which are summary statistics derived from dynamical systems theory and are calculable directly from observed data.

Dynamical systems theory states that as a stable system approaches a tipping point, the time taken to recover after a perturbation increases; ultimately diverging at the transition [4]. This phenomenon, known as critical slowing down, is expected to be observable in systems which are continually subject to shocks—whose origin can be either extrinsic (for instance, environmental fluctuations) or intrinsic (such as demographic noise). The effects of critical slowing down are manifest in the time series, leading to systematic changes in the summary statistics. For example, due to the persistence of perturbations the time series becomes increasingly correlated, which can be measured by the autocorrelation and correlation time. Because EWS are informed by generic properties derived from dynamical systems theory they have a key advantage over model fitting approaches: detailed calibration is not required. EWS have been applied to anticipate transitions in a range of complex systems, including ecosystem collapse in lakes and microbial communities [5,6], desertification in arid ecosystems [7] and changes in climate [8].

Identifying reliable warning signals is especially timely for emerging infectious diseases. Examples of pathogens with pandemic potential include influenza virus [9] and SARS-like coronaviruses [10]. Similarly, pathogens which have been successfully controlled can re-emerge, for example, pertussis [11].

Constructing validated models for emerging diseases is complicated in part by complexity, and also by virtue of the pathogen's novelty in a new host. Key epidemiological determinants, such as the infectious period, the mode of transmission, infectiousness and rate of spillover, are typically unknown or poorly quantified. This makes them a prime candidate for model-free approaches, such as EWS. To date, work on anticipating disease emergence has predominantly focused on specific systems and models, for instance, in studies on the effects of climate change on disease emergence [12], with a previous attempt at applying EWS to simulated data finding the approach to emergence difficult to detect [13].

The scenario considered in this paper is based on the slow emergence of a pathogen. This can occur for a variety of reasons: as a result of changing immunological landscape (increasing the susceptible pool—cf. monkeypox [14] or measles [15]), pathogen adaptation to new host [16], immune evasion [17,18] or long-term transient dynamics (the ‘honeymoon effect’ [19]). These mechanisms all lead to increasing inter-individual transmission of the pathogen—characterized by an increase in *R*_0_, a threshold quantity. The primary concern for disease containment is averting the tipping point which occurs when *R*_0_ = 1.

Formally, *R*_0_ is defined as the average number of secondary infections caused by an infectious individual [20]. From theory we know that if *R*_0_ < 1 transmission is subcritical and the pathogen is incapable of long-term persistence. Instead, continued circulation can only be sustained through repeated reintroduction from an external reservoir. In this regime, the transmission dynamics are characterized by what are known as ‘stuttering chains’ [21]. On the other hand, if *R*_0_ > 1 the disease will typically invade successfully and grow in a fully susceptible population, leading to a large-scale outbreak and possible endemicity [22]. Such dynamics are referred to as supercritical. It is this transition, from subcritical to supercritical, that we seek to anticipate.

The birth–death–immigration (BDI) process can be viewed as the simplest possible model of emerging diseases with direct transmission, modelling spillover of a pathogen from an external source, together with secondary chains of transmission fed by a large susceptible pool. A major benefit in using the BDI process is that it has a known analytical solution [23], which we use as the basis for the two approaches presented. For the first approach, we derive key metrics which may be used as EWS. The second approach uses a likelihood-based method to formally test emergence as a hypothesis.

As they are derived from a very generic model of disease emergence, the approaches are expected to have applicability across directly transmitted emerging disease systems. We test the robustness of measures to changes in model structure, simulating disease emergence using the SIS and SIRS compartmental models subject to varying immunology and demographics. Additionally, we comment on how the speed at which the transition is crossed affects the reliability of the two approaches, something which has been considered poorly understood in previous work on EWS [24].

## 2. Early-warning signals using the birth–death–immigration process

**Figure 1.**
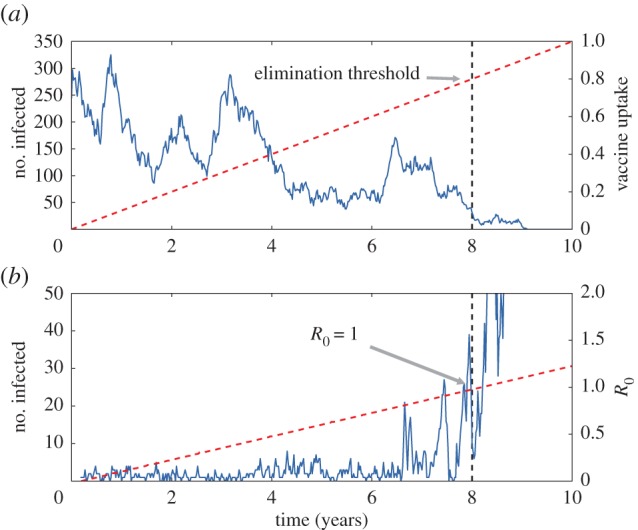
(*a*) Simulation of disease elimination through vaccination. At time *T* = 8 years the vaccine threshold for elimination is reached. Weekly data were generated using the SIR model with birth and death [20]. The mean infectious period 1/*γ* = 14 days, the death rate *μ* = 0.02 yr^−1^, *R*_0_ = *β*/(*γ* + *μ*) = 5 and the population size *N* = 4 × 10^5^. The birth rate of susceptible individuals is affected by vaccination via *ν* = *μ*(1 − *v*), where *v* is the vaccine uptake, which linearly increases from 0 to 1 over the 10 year period. The elimination threshold is passed when *v* = 1 − *R*^−1^_0_ = 0.8. (*b*) Simulation of disease emergence through increasing infectiousness. Simulations were performed using an SIS model with 1/*γ* = 7 days, *N* = 10^3^ and with *R*_0_ = *β*/*γ* increasing linearly from 0 to 1.25 over the 10 year period. Susceptible individuals acquire infection from external sources with *per capita* rate *ζ*/*N* = 7 × 10^−4^ d^−1^. All simulations in this paper are performed using the NRM algorithm [25,26]. (Online version in colour.)

Previous work exploring EWS for infectious disease dynamics has shown that performance depends on the direction the critical transition is traversed, with the two contrasting cases shown in figure 1 [13]. In studies employing a combination of analytical results and simulated data, EWS have successfully been identified for diseases approaching elimination—for instance, due to increasing vaccine uptake—where *R*_0_ approaches the critical transition from above [13,27]. The case of disease emergence has thus far proven more challenging [13].

This challenge can be understood from a theoretical perspective. The conventional theory of EWS, as detailed, for example, by Scheffer *et al.* [4] and used by O'Regan & Drake [13], relies on the presence of critical slowing down, presupposing that the system is at a stable fixed point and subject to exponentially decaying perturbations. When the number of infectious individuals is sufficiently large the transmission dynamics can be well approximated as a linearized system subject to Gaussian noise [28,29].

For endemic diseases prior to elimination the number of infected individuals is large enough for this approximation to be valid, and EWS perform well [13]. Conversely, for subcritical emerging diseases there are typically very few infected individuals present in the population. This means that the effects of demographic stochasticity feature strongly in the dynamics [29]. The stochastic dynamics are highly non-Gaussian and the conventional theory of critical slowing down is invalid. In the light of these findings, we instead develop EWS starting from an alternative model, the BDI process, which explicitly incorporates transmission between individuals. By starting from a model which captures the conditions preceding disease emergence, we show how EWS can be expected to behave for this class of transition, and assess their prospects as generic indicators.

The BDI process is one of the simplest models of a subcritical disease [23]. It neglects the effects of susceptible depletion, assuming that only a small fraction of the population are infected at any one time, and that the pool of susceptibles is replenished with sufficient speed. The BDI process focuses on three simple transitions: (i) an infected individual is introduced into the population, (ii) the infection can spread to other individuals with a rate proportional to the number of infected individuals present, and (iii) infected individuals may recover from the disease.

We assume that at a constant rate *ζ* individuals import the infection due to contact with external sources, for example a zoonotic reservoir. We focus on diseases which are emerging due to changes in the transmission rate of the disease, *β*(*t*). If there are *n* infected individuals present in the population, then the total force of infection is λ(*t*) = *β*(*t*)*n* + *ζ*, and the rate of recovery is *γn*. Throughout the paper we assume that changes in *β*(*t*) occur on a much slower time scale than the typical duration of an infection, 1/*γ*.

The transition rate *T*_*m*,*n*_ is defined as the probability per unit time of transitioning from a state with *n* infected individuals to a state with *m* infected individuals. The BDI process is a one-step stochastic process which consists of two possible transitions; for infectious diseases these are infection and recovery. Respectively, the transition rates for infection and recovery are2.1
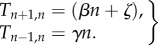
In the BDI process, the chain of transmission stemming from a particular introduced index case is given by a branching process [30]. A particular outbreak can be considered as a superposition of the separate chains of transmission caused by each introduced case during the outbreak [31]. The basic reproductive number is defined as the average number of secondary cases, *R*_0_ = *β*/*γ*, found using the offspring distribution of the associated branching process [30,32].

The probability of *n* individuals being infected at time *t* is *P*_*n*_(*t*). The change in probability with time is found by solving the master equation, a set of coupled linear differential equations built from the transition rates. For the BDI process,2.2
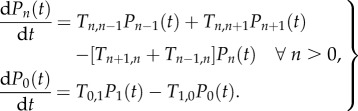
The advantage in considering such a simple model is that the master equation, which determines how the probability distribution of the number of infectious individuals changes in time, can be solved exactly without the need for any approximations [23]. This can be achieved through use of the moment generating function, 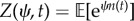. The variable *ψ* is used to find the moments and correlation functions of the stochastic process via differentiation, for example, the *i*th moment, 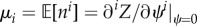. We find from the master equation that *Z*(*ψ*, *t*) solves the partial differential equation2.3
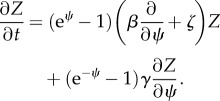
Further details of the master equation calculation are presented in appendix A.

Using that *R*_0_ = *β*/*γ*, the mean-field behaviour of the system solves d*μ*_1_/d*t* = (*R*_0_ − 1)*γμ*_1_ + *ζ*. If *R*_0_ > 1 then *μ*_1_ grows exponentially, whereas if *R*_0_ < 1 the disease persists at a low level, *μ*_1_ = (*ζ*/*γ*)/(1 − *R*_0_), sustained by immigration.

Table 1 shows the list of candidate EWS calculated from the moment generating function of the stationary BDI process which will be considered in this paper. The mean, variance, index of dispersion (also known as the variance to mean ratio), and correlation time all diverge as *R*_0_ → 1. The autocorrelation approaches one, whereas the coefficient of variation remains constant below the transition.

**Table 1.** Measurable quantities calculated from the BDI process. All expressions are valid for 0 ≤ *R*_0_ < 1. See equations (A 3), (A 4) and (A 7) for details.

early-warning signal	formula
mean	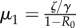
variance	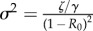
coefficient of variation	*σ*/*μ*_1_ = (*ζ*/*γ*)^−1/2^
index of dispersion	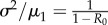
correlation time	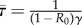
autocorrelation	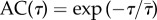

The correlation time gives a measure of the persistence of correlations. Its divergence as *R*_0_ → 1 implies that correlations persist for extremely long times, and is the mathematical definition of critical slowing down. Intuitively, critical slowing down can be understood by considering the lengths of transmission chains. Although on average each imported case will infect fewer than one individual (as *R*_0_ < 1), there is a possibility that a large chain of secondary cases is sparked, which then takes a long time to die out. As *R*_0_ increases, the probability of larger chains of transmission increases, in turn leading to an increasingly autocorrelated dynamics. The increased probability of larger chains of transmission also causes an increase in the mean and variance of the number of infected.

In addition to the EWS derived from the BDI process presented in table 1 we also study two signals from information theory: the Kolmogorov complexity, and the Shannon entropy [33]. The Kolmogorov complexity is a measure whose origin lies in the length of the minimal computer program needed to reproduce the sequence of data, with longer computer programs implying more complex data [33–35]. The increased correlation near the transition may therefore result in a decrease in complexity, which can serve as an EWS. The Shannon entropy is a measure of the information content of the time series. As *R*_0_ approaches one, the probability of rare large outbreaks increases, which suggests the entropy will also increase near the transition. For details of how these two measures are computed, see appendix B.

Two simplifying assumptions are implicit in the model. Firstly, the BDI process neglects susceptible depletion. Secondly, the speed with which *β* changes is ignored, assuming that it varies infinitely slowly. The effects of relaxing the first of these assumptions will be investigated in the next section. Disease emergence over faster time scales will be investigated in §5.

## 3. Comparison with models featuring susceptible depletion

For EWS to be useful they must be robust to changes in model structure, and not just specific to the BDI process. Using results from simulations of common disease models, in this section we explore the sensitivity of the EWS to a range of demographic and immunological properties.

**Figure 2.**
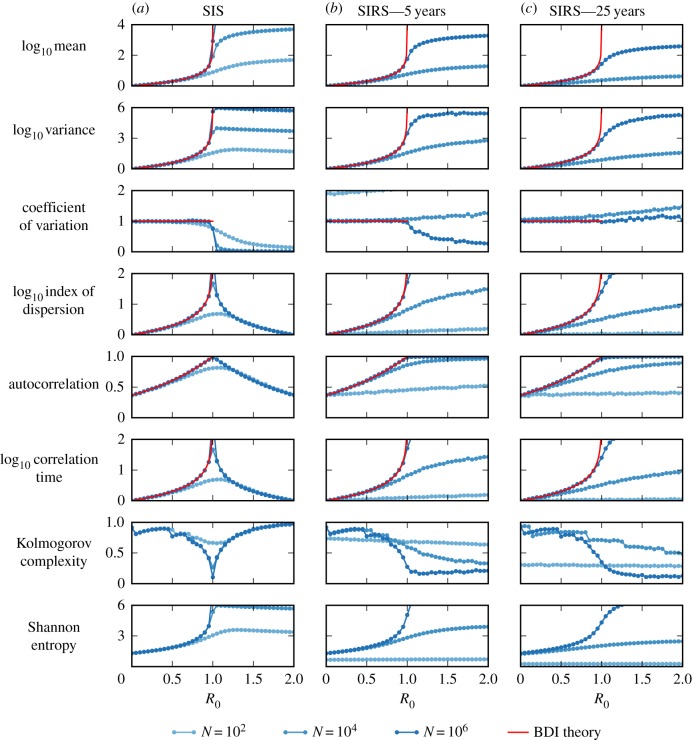
EWS for the stationary SIS and SIRS models. Red lines correspond to the theoretical results found from the BDI model (table 1). Symbols depict the results of simulations with the duration of immunity as indicated in the figure. Independent simulations are performed for each value of *R*_0_. EWS are calculated by time averaging over 4 × 10^3^ weeks of data (see appendix D). The force of infection in the SIS and SIRS models is λ = (*βn* + *ζ*)/*N*, where *n* is the number of infected and *N* the total population size. The remaining model parameters are *γ* = 1 and *ζ* = 1 week^−1^. The autocorrelation is calculated at lag one week, AC(1). (Online version in colour.)

The SIS model is a model of infectious disease spread in which individuals move from the susceptible class to the infected class upon infection, and then return to the susceptible class when they cease being infectious. The total population size is assumed constant. The SIRS model is similar to the SIS model, but in addition the pathogen confers temporary immunity to reinfection, which wanes at rate *ρ* [20]. The dynamics of both models can be represented using a master equation, as with the BDI process. Exact numerical realizations of these models can be obtained using the next reaction method (NRM) algorithm [25,26]. The candidate EWS for the SIS and SIRS models are shown in figure 2. The results for the BDI process listed in table 1 are also plotted.

The only additional parameter in the SIS model is the population size, *N*, allowing the effects of susceptible depletion on EWS to be studied in isolation (figure 2*a*). We find that typically, the effect of reducing the population size is to decrease the responsiveness of a signal to increases in *R*_0_. The mean and variance always increase prior to the transition; however, the magnitude of the increase is reduced. The value of *R*_0_ for which the variance is maximized is seen to increase. A similar effect is also observed for the index of dispersion, autocorrelation and correlation time. These three EWS all peak around *R*_0_ = 1 for large population sizes, but for *N* = 10^2^ there is a noticeable shift in the peak position and height to the right. For the coefficient of variation, decreasing the population size smooths the abrupt change in value at *R*_0_ = 1. The Kolmogorov complexity falls as the transition is approached, in line with the increased correlation: if there is a fluctuation above (or below) zero within the detrended data it is more likely to be sustained in further data points. Regular sequences allow for increased use of the copy operation (see appendix B), reducing the complexity of the time series. Perhaps counterintuitively, the complexity of the time series is lower near the transition for larger populations. The entropy has a very similar behaviour to the variance, peaking in the vicinity of the transition. Overall, the results from the BDI process appear to provide an upper bound on the values of the EWS in the SIS model.

Figure 2*b*,*c* shows results from the SIRS where the mean duration of immunity, 1/*ρ*, is 5 and 25 years. For the smallest population size, *N* = 10^2^, the mean number of infected individuals is less than one, irrespective of *R*_0_. As might be expected, EWS vary little with *R*_0_ and do not display the trends observed for the BDI process. For larger population sizes, the behaviour of some EWS above the transition is markedly different from the SIS model, for example, the autocorrelation remains close to one and the complexity decreases with *R*_0_. Below the transition, the opposite is the case for *N* > 10^2^, with the EWS having similar behaviour to the BDI process. For a given population size, the responsiveness of the EWS decreases with immune duration, cf. the similarity in the index of dispersion and Kolmogorov complexity for *N* = 10^4^ in the SIS model and *N* = 10^6^ in the SIRS model. Apart from the coefficient of variation, the BDI process results again provide upper bounds on the EWS, as with the SIS model.

Below the transition, decreasing the population size and increasing the immune duration reduce the responsiveness of the EWS. This can be understood by returning to the theory of critical slowing down. As the susceptible pool is depleted the probability of further disease transmission is diminished, reducing the probability of extremely long chains of transmission. The impact of susceptible depletion is greater for smaller population sizes. Increasing the duration of immunity means individuals who acquire the infection are absent from the susceptible pool for longer, magnifying the impact of susceptible depletion. The longer the duration of immunity the larger the susceptible pool required for agreement with the BDI results.

Below the transition each EWS behaves similarly regardless of the model, provided that the population size is large. This independence of model structure supports the general applicability of BDI results to more complex dynamics. We note that there is a large distinction in behaviour between models above the transition; for instance the coefficient of variation strictly decreases for the SIS model, whereas for the SIRS model it may decrease, increase or remain approximately flat depending on the model parameters. The generality of the EWS is strictly for disease emergence.

## 4. Cox's *δ*: a likelihood-ratio test

An alternative model-specific approach to detecting critical transitions has been proposed by Boettiger and Hastings, focusing on anticipating the loss of stability of fixed points [24]. They propose likelihood-ratio testing, using a statistic they refer to as Cox's *δ*. The test determines whether the dynamics are approaching a transition or are instead stationary. Each hypothesis is represented by a model, and a maximum-likelihood estimate (MLE) for the data is found. The value of Cox's *δ* provides a measure of how much disease emergence is favoured. The procedure by which Cox's *δ* is calculated also allows for an estimation of the time remaining until the transition is reached.

Boettiger and Hastings assume that the Ornstein–Uhlenbeck (OU) process is an appropriate model for the dynamics. The OU process is a continuous-time stochastic process in which there is deterministic reversion to the mean, with Gaussian white noise perturbing the system [28]. It has a known mathematical expression for the likelihood of a time series, which Boetigger and Hastings use to efficiently calculate Cox's *δ* and thereby determine whether the system is approaching a transition [24].

A barrier to using Cox's *δ* as a method for anticipating disease emergence is this reliance on the OU process to calculate the likelihood. Dynamically, the OU process does not constrain the number of infected individuals to be a non-negative integer. When there are a small number of infected individuals present in the data, this means biologically impossible paths are included in the likelihood calculation. The problem can be overcome by instead using the BDI process, which presents a more biologically plausible model of disease emergence.

An exact solution to the transition probability exists for the BDI process from which the likelihood of a time series can be constructed (see appendix C). We assume *R*_0_(*t*) can be decomposed into two parts, a baseline which is set as *R*_0_(0) and a linear trend, Δ*R*_0_, i.e.4.1
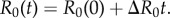
For the test hypothesis (the disease is emerging), the MLE is calculated from a likelihood surface allowing both of these parameters to vary. In calculating the MLE for the null hypothesis (no emergence), we fix Δ*R*_0_ = 0, still allowing *R*_0_(0) to vary. For an observed time series {*n*}^*T*^_*t*=0_, the log-likelihood of the MLE for the test model is4.2
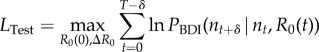
and for the null model is4.3
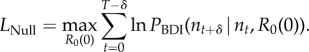
The expressions for *P*_BDI_ are presented in appendix C.

Cox's *δ* statistic is defined as twice the difference in the log-likelihoods of the MLEs for the two nested models,4.4
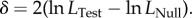
Because the models are nested *δ*≥0. Increasingly positive *δ* implies stronger support for the test model over the null model. A *δ* > 2 is taken as the criterion for concluding that the disease is emerging, on the basis of the AIC score [24].

**Figure 3.**
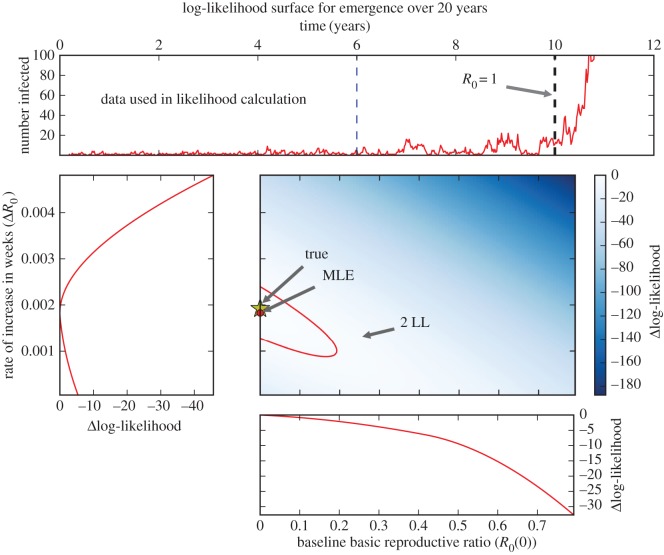
Estimating the rate of emergence using maximum-likelihood methods. Simulation generated using an SIS model with *R*_0_(*t*) = *R*_0_(0) + Δ*R*_0_*t* increasing linearly from 0 to 1.2 over 12 years. Data are output weekly. The likelihood surface is calculated using only data from the first 6 years for parameters on a 100 × 100 grid of uniformly spaced data points with Δ*R*_0_(0) ∈ [0, 0.008] and *R*_0_(0) ∈ [0, 0.8]. The gold star indicates the location of the true parameter set and the red dot indicates the parameters corresponding to the maximum-likelihood estimate (MLE). Simulation performed using the modified next reaction method [26]. The remaining model parameters are fixed to their true values to reduce computation time; 1/*γ* = 1 week, *ζ* = 1 week^−1^ and *N* = 10^4^ individuals. The MLE is that *R*_0_(0) = 0.00 and Δ*R*_0_ = 1.83 × 10^−3^ week^−1^. The true values are *R*_0_(0) = 0.00 and Δ*R*_0_ = 1.92 × 10^−3^ week^−1^. Side panels show the likelihood profiles. (Online version in colour.)

Figure 3 shows the log-likelihood surface calculated using the BDI process for a time series generated using the SIS model. The MLE of the test model is *R*_0_(0) = 0.00 and Δ*R*_0_ = 1.83 × 10^−3^ week^−1^. The critical transition is predicted to occur after 10.52 years, very close to the true value of 10 years. Cox's *δ* statistic is 10.78, implying that the hypothesis of disease emergence is statistically supported [24]. One caveat is that this strong result has been achieved under idealized model conditions, assuming knowledge of the rates of immigration and recovery, and calculated using a long sequence of data (312 weeks of data). In the following section, we explore further the reliability of Cox's *δ* at anticipating transitions and compare with the EWS.

## 5. Impact of time scale on reliability of predictors

**Figure 4.**
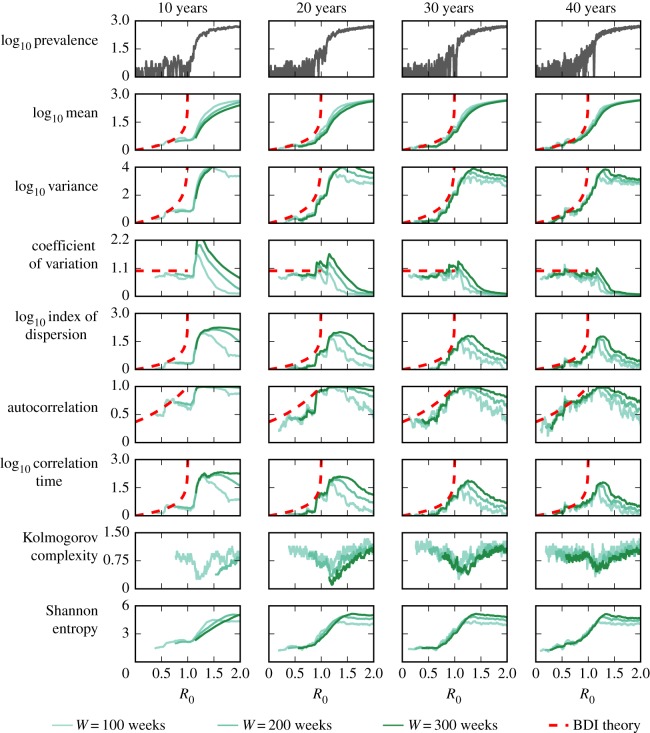
Performance of candidate EWS for various window sizes and transition speeds. Simulations are performed using an SIS model. Window sizes are 100, 200 and 300 weeks. The population consists of 1000 individuals, all other parameters are the same as in figure 2. Red lines correspond to the stationary results for the BDI model, green lines to simulated time series. EWS are calculated from the number of infected individuals in the population, data output weekly. The autocorrelation is calculated at lag one week, AC(1). (Online version in colour.)

**Figure 5.**
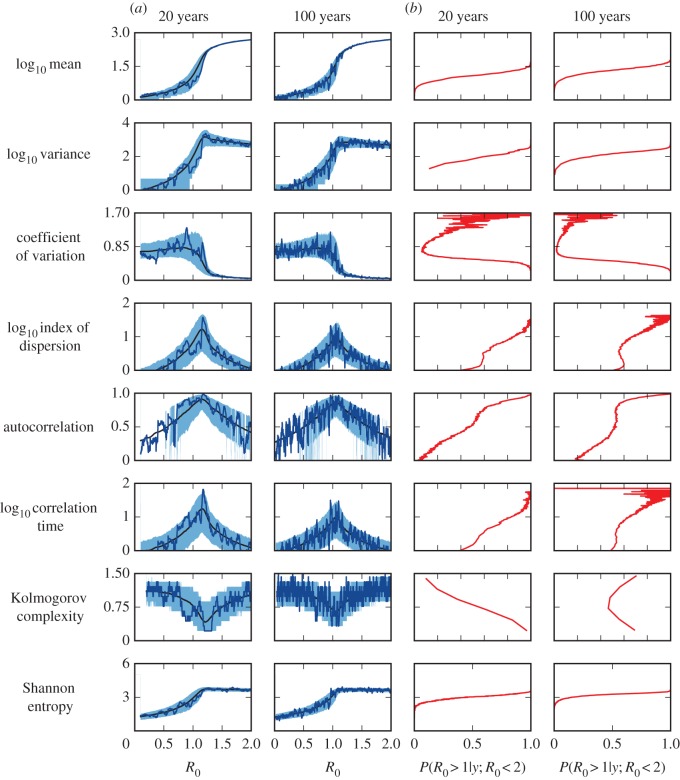
(*a*) 95% CIs of EWS for the SIS model with *N* = 1000. *R*_0_ increases linearly from 0 to 2. The total simulation time is *T* = 20 years and *T* = 100 years. Data are output weekly, EWS are calculated using a window size *W* = 52 weeks for both columns. The average of the replicates is shown in black and a sample replicate is shown in dark blue. (*b*) Numerical estimate of the probability that *R*_0_ > 1, given an observed value of an EWS. The noisiness in the estimate for more extreme values of the EWS is due to the infrequency of these events. (Online version in colour.)

**Figure 6.**
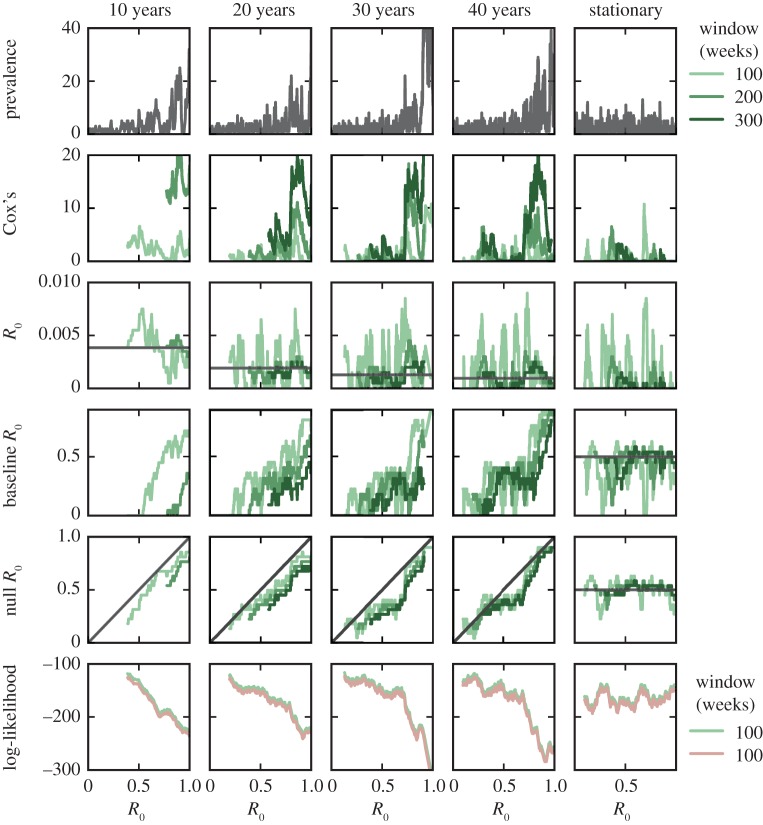
Cox's *δ* calculated using the BDI process solution. The time-series data are the same as used in figure 5, with the additional stationary time series generated by running the SIS model for 40 years with *R*_0_ = 0.5. Model parameters are *N* = 1000, *γ* = 1 and *ζ* = 1. Time to emergence is given as a fraction of the initial time to emergence. In the bottom row, the log-likelihood is shown for the test (green) and null (red) models for window size *W* = 100 weeks. (Online version in colour.)

**Figure 7.**
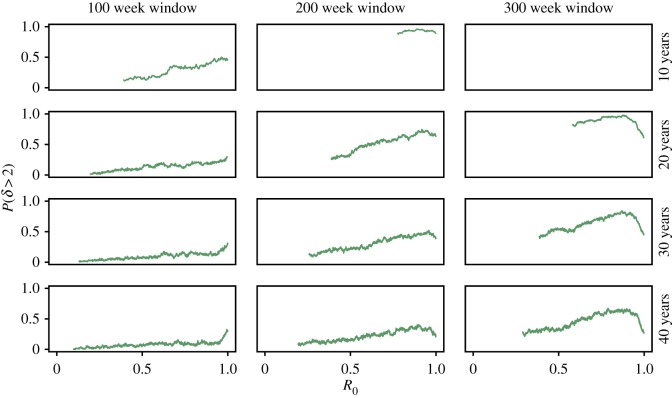
Probability that Cox's *δ* > 2, calculated from 200 replicates. Cox's *δ* computed using the likelihood-ratio test and BDI process solution, see §4. Fixed model parameters are *N* = 1000, *γ* = 1 and *ζ* = 1. (Online version in colour.)

**Figure 8.**
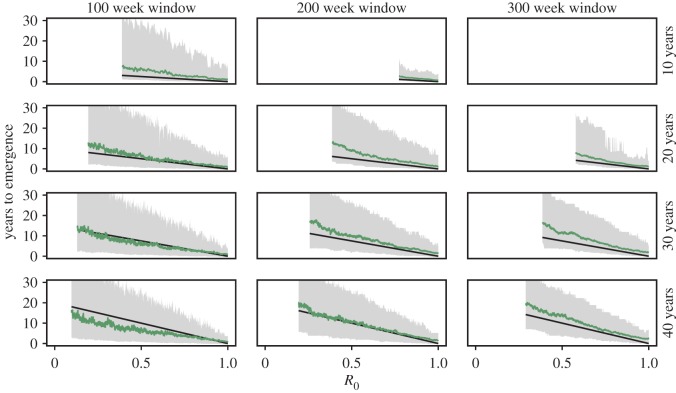
Mean and 95% CIs of the remaining time to emergence. Time to emergence calculated using the MLE, *T*_e_ = *W* + (1 − *R*_0_(0))/Δ*R*_0_, where *W* is the window size. True value of the time to emergence shown in black. Calculated using the same data as in figure 7. (Online version in colour.)

In §3, we presented results for stationary dynamics, appropriate for a disease emerging over an extremely long time scale. In practice, we are interested in anticipating disease emergence over faster time scales, as shown in figure 3. In this section, we investigate how the EWS and Cox's *δ* perform as indicators of disease emergence. Results for the EWS are shown in figures 4 and 5, and results for Cox's *δ* are shown in figures 6, 7 and 8.

Figure 4 shows the EWS calculated for the SIS model, with the stationary BDI process results also shown for comparison. The EWS are calculated from the time-series data; for simplicity, we use unweighted moving averages (see appendix D). The time series were generated by an SIS model with linearly increasing *R*_0_; from 0 to 2 over *T* = 10, 20, 30 and 40 years. The moving window average is calculated using three window sizes: *W* = 100, 200 and 300 weeks.

As shown in figure 4, all EWS bar one have a clear response to the approaching transition as long as the time scale is longer than 10 years (*T* > 10). For instance, the variance increases for all window sizes and speeds prior to *R*_0_ = 1, in line with predictions. The exception is the coefficient of variation, which performs poorly as an indicator of transitions, remaining largely unchanged until *R*_0_ > 1. It is also highly sensitive to the choice of window size, the sharp peak following the transition observed for *T* = 10 and 20 years increases with window size. The autocorrelation performs particularly well, reaching AC(1) > 0.85 for *T* = 20, 30 and 40 years. It does not appear particularly sensitive to window size below the transition.

As the speed of emergence is increased, the lag behind the BDI process results increases. Overall, we see that the transition speed has three notable effects which adversely impact all EWS. The first effect is dynamical: the incidence level is continually responding to the changing equilibrium resulting from increasing *R*_0_, leading to a lag between the values of the EWS calculated at equilibrium and those observed in the time series. Combined with demographic stochasticity, this leads to an apparent ‘bifurcation delay’ as the transition is traversed [36,37].

The second effect arises in computing the EWS. The theoretical results for the EWS are calculated by finding the moments and autocorrelation of a stochastic process. Owing to the absence of multiple replications, these are calculated over a moving window [4]. However, when the disease emerges over a finite time scale, the process is non-stationary, and therefore also non-ergodic, due to changing *R*_0_(*t*). In practice, this means that averages are calculated using data points which were generated at different values of *R*_0_; for example, if *T* = 10 years and *W* = 100 weeks, the difference in *R*_0_ between the start and end of the window is 0.38. This reduces the responsiveness of the EWS, also contributing to the lag behind the BDI process results. Furthermore, there can be other artifacts arising from non-ergodicity, for instance, increased variance of the data. Increasing both the window size and transition speed exacerbates these effects due to the larger range in values of *R*_0_.

Thirdly, there is Monte Carlo error due to the limited number of data points used. This is manifest in the stochastic variation of the observed EWS as *R*_0_ changes. This is particularly apparent in the first column of figure 4, where a chance drop in prevalence immediately prior to *R*_0_ = 1 leads to a failure of most EWS to behave as theory predicts. Increasing the size of the window has the effect of reducing this error (evidenced by the smoother curves for *W* = 300), however, at the expense of the signal's responsiveness, as detailed above.

Given the existence of Monte Carlo error, to quantify the performance of the EWS further, we compute 90% CIs for the EWS for disease emergence over 20 years and over 100 years using a window size of 52 weeks (figure 5*a*). A measure of how effective an EWS is at detecting emergence is the range in *R*_0_ over which a specific reading of an EWS lies within the 90% CI. A wide range means that the EWS gives little information about the value of *R*_0_. For the variance, a reading of *σ*^2^ = 40 falls within the 90% CI for 0.8 ≲ *R*_0_ ≲ 1.1, implying a strong chance that the system is near the transition. A reading of *K* = 0.8 for the Kolmogorov complexity falls within the 90% CI for all values of *R*_0_ shown, implying a poor predictive power.

To quantify this further, figure 5*b* shows an estimate of probability that *R*_0_ > 1 given a particular reading of an EWS, *y* (appendix E provides details on how this estimate is calculated). The sensitivity of *P*(*R*_0_ > 1 | *y*) to changes in *y* depends heavily on the EWS. Large values for the mean, variance and entropy strongly indicate that transmission of the pathogen is supercritical. On the other hand, for EWS which peak near *R*_0_ = 1, such as the index of dispersion, autocorrelation and correlation time, there is low indication whether the disease is supercritical or not, unless the observed value is especially large.

Because of their narrow confidence intervals, the variance and entropy are exquisitely sensitive, with *P*(*R*_0_ > 1 | *y*) increasing from near 0 to 1 over a small range of *y*. Others, such as the index of dispersion and autocorrelation, have an intermediary range of values of *y* where *P*(*R*_0_ > 1 | *y*) ≈ 0.5.

We see a trade-off between how reliable an EWS is at classifying whether *R*_0_ > 1 and the range of values of *R*_0_ over which *P*(*R*_0_ > 1 | *y*) notably increases. A practical conclusion from this is that sequential readings of an EWS are needed to ascertain whether a disease is emerging.

Using a moving window, in figure 6 we show Cox's *δ* calculated for the same set of time series studied in figure 4. As the transition is approached Cox's *δ* typically increases in significance, with large responses for window sizes *W* = 200 and 300 weeks. There are spurious increases in Cox's *δ* for the stationary time series, due to the intrinsic variability of the dynamics, but these are dampened by using a larger window.

To test its reliability as an indicator of disease emergence, figure 7 shows the probability that *δ* > 2 as the transition is approached.

Large window sizes lead to an improved performance, as does a faster speed of emergence. For faster speeds of emergence (*T* ≤ 20 years) and larger windows (*W* ≥ 200 weeks), Cox's *δ* performs well, with *P*(*δ* > 2) > 0.75 prior to the transition. From figure 7, the recommendation is to use the largest window size feasible given the data.

The effect of the speed of emergence and the window size on the performance of Cox's *δ* is more subtle than for the EWS shown in figure 4. For EWS, the performance improves when the disease emerges at a slower rate due to the smaller change in *R*_0_ between the start and end of the window. The opposite is the case for Cox's *δ*. Instead, slower emergence reduces the difference in likelihood between the emerging and non-emerging models, reducing the power of Cox's *δ*.

From the estimates of the baseline *R*_0_ and Δ*R*_0_ the time to emergence can be estimated (figure 8). The mean estimated time to emergence generally does agree well with the true value, with typically marginally better agreement for smaller window sizes. However, the 95% CIs are large, in line with the variation in the MLE of Δ*R*_0_ seen in figure 6. Increasing the window size reduces to the confidence intervals, with a more pronounced reduction in the probability of underestimating the time to emergence, cf. the difference between *W* = 100 and 300 weeks for *T* = 20 years. With sufficiently large window size given the speed of emergence, the estimated time to emergence can be taken as an overestimate.

## 6. Discussion/conclusion

This work is intended to provide a theoretical base for the development of methods to anticipate disease emergence. We have presented two parallel approaches by which this may be accomplished. The first approach is to extend the burgeoning literature on EWS, reformulating them in a manner appropriate for emerging diseases. The second approach makes use of Cox's *δ*, a likelihood-ratio test for emergence. For both approaches, the application to emerging diseases is achieved through use of the BDI process, a well-understood model from the theory of stochastic processes.

Both the EWS and Cox's *δ* perform best when calculated using a large number of data points as this reduces statistical uncertainty. The time scales imposed by real-world disease emergence likely mean that available data are more limited. We therefore apply both approaches to weekly time series data of diseases emerging over a range of different intervals. We find that—apart from the coefficient of variation—all EWS undergo similar behaviour regardless of the time scale of emergence. More advanced time averaging methods may be needed for very fast emergence to detect a strong signal. Although larger window sizes do reduce uncertainty they are typically not necessary, with *W* = 100 weeks being sufficient. Cox's *δ* performs best with the largest window size possible so that there is the largest possible difference in *R*_0_ between the start and end of the window.

Overall we conclude that the two methods are complementary, performing best under differing conditions. Cox's *δ* reliably detects emergence over fast time scales, where the null hypothesis is strongly disfavoured. EWS perform best at detecting long term trends in the time series, where there is a more gradual approach to the epidemic transition. Additionally, our results favour the use of some EWS over others. The behaviour of the coefficient of variation and Kolmogorov complexity prior to the transition means they both perform poorly as indicators of emergence. The remaining EWS (the mean, variance, index of dispersion, autocorrelation, correlation time and entropy) have a behaviour which is resiliant to parameter changes and reliable in the face of stochasticity, making them strong candidate EWS.

By grounding our results in theory, rather than a detailed model for a particular disease we expect our results will have applicability to a wide range of emerging infectious diseases. This generality is of particular importance for emerging infectious diseases, where there are likely many unknowns regarding transmission of the pathogen.

The key simplifying assumption of the BDI process which makes it mathematically solvable is that it neglects the impact of disease transmission on the availability of susceptible individuals. In reality, infection diminishes the size of the susceptible pool and can also confer immunity to reinfection, delaying the individual's return to susceptibility. Our results show how these demographic and immunological considerations impact on the performance of EWS. Although the signals' strengths are reduced, for all parameters considered the performance is still informative for a population of 10^6^ individuals.

Our work is not the first to investigate the signature of criticality prior to the epidemic transition; in particular, we note the work of Jansen and collaborators [15]. These previous works seek to identify the approach to the critical transition through changes in the tail of the outbreak size distribution [15,38]. In contrast, both approaches in this paper are based around summary statistics. This has advantages: the sizes of individual distinct outbreaks can be difficult to identify, a large number of outbreaks are required to accurately reconstruct the tail of the outbreak size distribution, and summary statistics calculated using moving windows clearly display temporal variation in the dynamics.

The SIS and SIRS models considered in this paper have only one susceptible and one infected class, assuming all individuals are equally infectious and interact homogeneously. Risk structure has been shown to significantly change the necessary conditions for epidemics, with *R*_0_ = 1 being an underestimate for the epidemic threshold [39]. For diseases emerging in populations structured as many loosely connected smaller community units, such as Ebola in sub-Saharan Africa, this may significantly impact on predictability. *R*_0_ is a measure based on the average context; however heterogeneities in the contact structure of individuals can lead to instances of superspreading [22], particularly relevant for sexually transmitted diseases and something not captured in the BDI process. The large variations in transmission rates reduce the worth of *R*_0_ as a measure, and a universal increase in *R*_0_ is unlikely to be the driver of emergence. Superspreading events have been identified for SARS and more recently for MERS [21,40]. In addition, there is the challenge of incomplete and unreliable data, modelled through explicit inclusion of a reporting process [41]. Further work is ongoing studying EWS and Cox's *δ* for these dynamics.

This work lays theoretical foundations, but more research is needed to develop actionable technologies applicable to actual disease data. For instance, improved reporting would lead to an increase in the mean number of case reports, but not an increased autocorrelation. A simultaneous increase in both presents stronger evidence of disease emergence. The development of ways in which readings of multiple EWS and also Cox's *δ* can be combined and leveraged to improve the quality of predictions is therefore desirable. One motivation in studying EWS is to ultimately develop comprehensive EWS software packages. These packages would be plug and play ready: public health practitioners could use them to give quantifiable information about the risk of a critical transition and disease emergence in a particular context.

As a final note, while this paper has focused on anticipating infectious disease emergence this does not limit the applicability of our methods. Emerging diseases are an example of an invasion process, where a new class of individual attempts to establish itself in a population. Other examples are found across the biological sciences. The BDI process is an appropriate model for a large range of invasion processes. We propose that the methods in this paper will have a similarly general status for anticipating successful invasion. Our work, therefore, opens up a new class of transitions which it may be possible to anticipate.

## Data accessibility

All code and data are available at Dryad: http://dx.doi.org/10.5061/dryad.7kt3h [42].

## Appendix A. Critical slowing down for the stationary birth–death–immigration process

The master equation for the BDI process, equation (2.3), can be solved both when *R*_0_, *γ* and *ζ* are constant or when they are functions of time [23]. For mathematical simplicity, we will focus on the case in which the disease is emerging over a long time scale relative to the infectious period. Under these conditions, we can find exact theoretical values for the EWS. If there are *y* infectious individuals initially present then *Z*(*ψ*, 0) = e^*ψy*^ and equation (2.3) has the solution [23]

A 1

with *A*(*ψ*) = (1 − *R*_0_ e^*ψ*^) and *B*(*ψ*, *t*) = e^(*R*_0_^^−1)*γt*^(e^*ψ*^ − 1).

We are interested in dynamics prior to the emergence of disease so restrict ourselves to considering *R*_0_ < 1. As *τ* → ∞, we see that *B*(*ψ*, *t*) → 0. The generating function converges onto the stationary solution

A 2

for any number of initial infected. This is the moment generating function of a negative binomial distribution with mean and variance

A 3

and

A 4

Both equation (A 3) and equation (A 4) diverge as *R*_0_ → 1. Using equations (A 1) and (A 2), we can calculate the correlation function in the stationary state. The conditional mean, , is

A 5

and therefore the correlation function  is

A 6

The autocorrelation, defined for a stationary process as AC(*τ*) = (*C*(*τ*) − *μ*^2^_1_)/*σ*^2^, is

A 7

As the critical transition is approached, the correlation time  diverges. This is the hallmark of critical slowing down.

## Appendix B. Shannon entropy and Kolmogorov complexity

The Shannon entropy is a measure of the information content of a random variable, and is defined as  [33]. For time series data, an approximation to the probability distribution over *n* is calculated numerically via , where *n*(*s*) is the number of infected individuals present at time *s* and _*n*=*n*(*s*)_ is the indicator function, equal to one if *n* = *n*(*s*) and zero otherwise. As mentioned above, when the process is non-stationary this method is inexact.

Another information theoretic measure is the Kolmogorov complexity [33]. The Kolmogorov complexity of a sequence of data is the shortest computer algorithm required to reconstruct it. A quantification of the Kolmogorov complexity was proposed by Kaspar & Schuster [34]. Before applying their algorithm the data are converted to sequence of bits, 0 or 1. Their algorithm proceeds by considering two operations: ‘copy’ and ‘insert’. Each operation adds one to the complexity measure. The Kolmogorov complexity is the minimum number of operations required to reconstruct the data sequence. For further details, see [34]. The specific algorithm used to calculate the Kolmogorov complexity can be found in [35]. As with the other EWS, we calculate the Kolmogorov complexity over a moving window. To convert the time series to a sequence of bits, we perform a linear detrending over the data within the window. The residual time series is discretized into 1 whenever the residual is larger than zero, and 0 otherwise.

## Appendix C. Transition probability distribution and likelihood

The generating function given in equation (A 1) can also be used to find the the probability of there being *n*_*t*+*δ*_ infected at time *t* + *δ* given *n*_*t*_ infected at time *t* [28]. Assuming that *R*_0_ does not vary significantly between time *t* and *t* + *δ*, the transition probability for the BDI process is

C 1

where

C 2

and

C 3

with *ρ* = [1 − AC(*δ*)]/[1 − *R*_0_AC(*δ*)] and *p* = *μ*_1_/(*μ*_1_ + *ζ*/*γ*) = 1/(2 − *R*_0_). Given a time series {*n*_*t*_}^*T*^_*t*=0_, the likelihood of the data for a given rate of emergence, *R*_0_(*t*) = *R*_0_(0) + Δ*R*_0_*t*, is

C 4

The MLE for (*R*_0_(0), Δ*R*_0_) is given by  .

## Appendix D. Calculating early-warning signals from single time-series data

If the BDI process is stationary then the process is ergodic, allowing expectation values to be calculated from a time series by time averaging. For example, the correlation function is

D 1

The sum is performed over integer multiples of the timestep *δ*, with the approximation becoming exact as *W* → ∞. Although not formally equivalent, and therefore potentially introducing errors (see the discussion in §5), we will also use this approach to calculate expectation values for non-stationary processes, in line with the early-warnings literature.

## Appendix E. Estimating the probability of supercriticality

Suppose we observe that EWS *Θ* has a value *y*. We would like to know the probability that *R*_0_ > 1 given this observation, *P*(*R*_0_ > 1 | *Θ* = *y*).

Monte Carlo sampling allows for the estimation of *P*(*Θ* = *y* | *R*_0_ = *r*). Through use of Bayes' theorem,

E 1

Assuming a prior *P*(*R*_0_ = *r*) = 1/*Λ* if *r* ∈ [0, *Λ*] and 0 otherwise,

E 2

Finally, *P*(*R*_0_ > 1 | *Θ* = *y*) can be found numerically by binning recorded values of *Θ* and approximating the integrals as summations over all bins.
